# Case Report: Giant myxosarcoma involving both atria

**DOI:** 10.3389/fsurg.2026.1765767

**Published:** 2026-04-30

**Authors:** Jianbo Xue, Yiming Ni, Jinyu Zheng, Xianshuai Li

**Affiliations:** 1 Department of Cardiothoracic Surgery, Jinhua Municipal Central Hospital, Affiliated Jinhua Hospital of Zhejiang University School of Medicine, Jinhua, Zhejiang Province, China; 2 Department of Cardiovascular Surgery, The First Affiliated Hospital of Zhejiang University School of Medicine, Hangzhou, Zhejiang Province, China

**Keywords:** atrialseptal occluder, bilateral atrial tumor, malignant cardiac tumor, myxosarcoma, rapid postoperative recurrence

## Abstract

**Background** Primary cardiac tumors are uncommon, with an incidence rate ranging from 0.001‰ to 0.3‰. Among cardiac tumors, 75% are benign and 25% are malignant. Approximately 50% of benign cardiac tumors are myxomas, while 75% of malignant cardiac tumors are sarcomas. Compared with myxomas, malignant cardiac tumors have a poorer prognosis. Cardiac myxosarcomas often have an insidious onset in young and middle-aged patients, with an overall survival period of 6 to 12 months. Similar to soft tissue tumors, cardiac sarcomas also consist of a range of histological subtypes. Among these, cardiac myxosarcomas are extremely rare, with only a few case reports documented in domestic and international literature to date. Due to similarities in imaging findings, they are often diagnosed as myxomas preoperatively, which may lead to insufficient resection during surgery. Owing to similarities in gross and histological features, coupled with the rarity of such cases, a small number of cases are misdiagnosed as myxomas in postoperative pathological examinations, and a definitive diagnosis is only made upon recurrence. This paper reports a case of a 23-year-old male patient with congenital heart disease and atrial septal defect, who had a history of interventional occlusion for atrial septal defect 12 years ago. The patient was admitted to the hospital for a rare giant myxosarcoma involving both atria, manifesting as rapidly progressive hemodynamic compromise. He underwent surgical resection of the cardiac tumor, and the pathological diagnosis was cardiac myxosarcoma. The tumor demonstrated highly malignant biological behavior. Rapid postoperative recurrence occurred, resulting in a short survival period. The patient eventually died of vena cava obstruction, heart failure and multiple organ failure secondary to tumor recurrence.

## 1 Introduction

Primary cardiac tumors represent a rare clinical entity. Since the 1970s, advancements in echocardiographic diagnostic techniques have facilitated the widespread implementation of surgical interventions for cardiac tumors. Benign cardiac tumors typically yield favorable outcomes following surgical treatment; in contrast, malignant cardiac tumors are extremely rare, and radical surgical resection is often unattainable. These malignant lesions are associated with poor postoperative prognoses, high rates of recurrence and metastasis, and most patients succumb to the disease within a short period after surgery (1). This paper reports a rare case of cardiac myxosarcoma, providing a reference for the diagnosis and treatment of similar cases.

## 2 Case report

**Figure 1:**
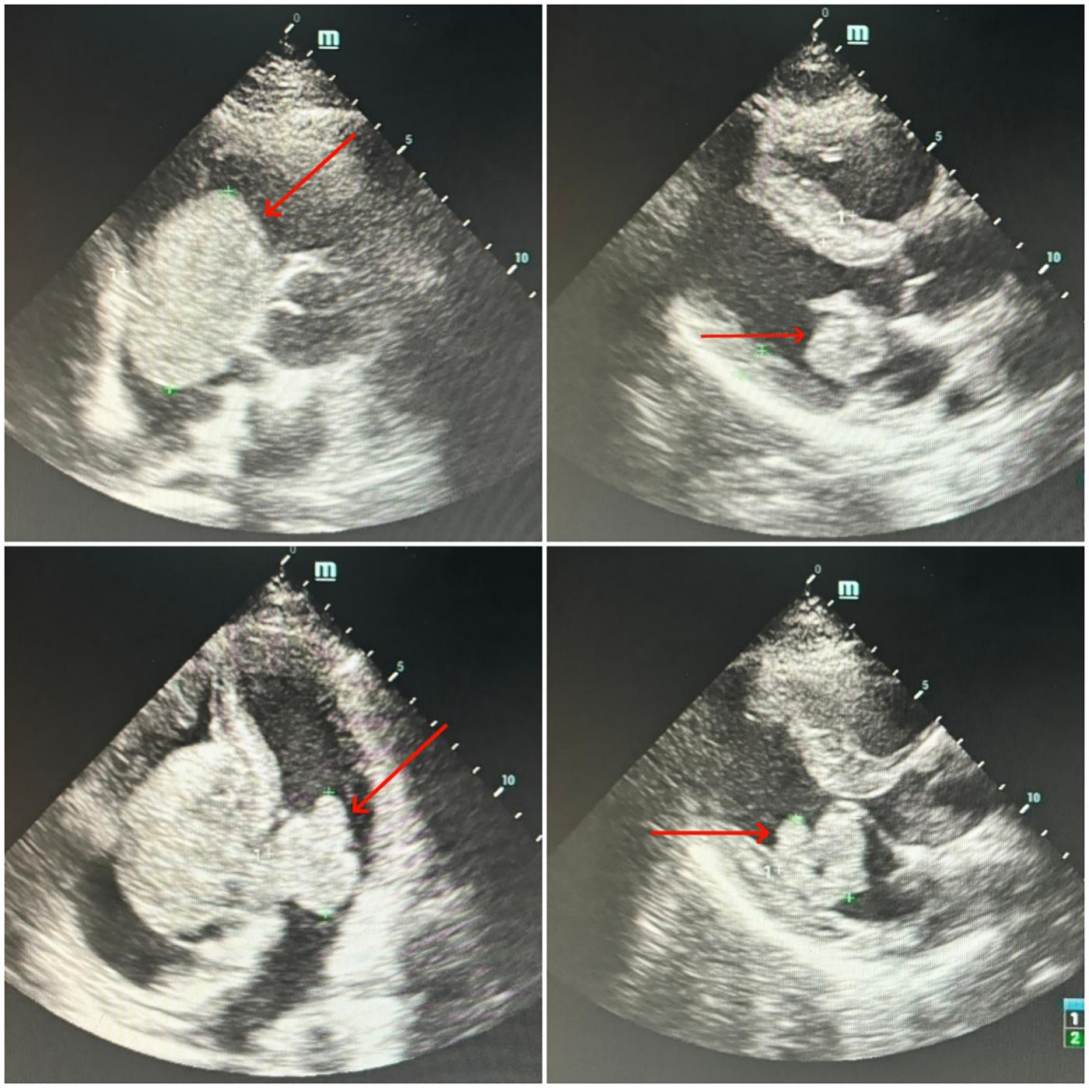
Echocardiography demonstrated giant hyperechoic masses occupying both the left and right cardiac cavities.

**Figure 2:**
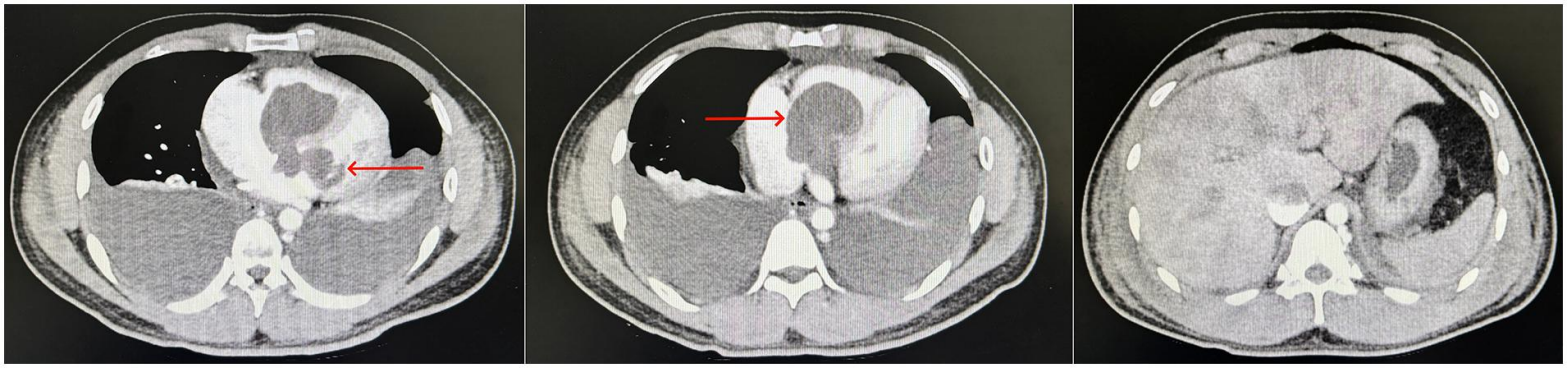
Giant space-occupying lesions were observed in both the left and right cardiac cavities, accompanied by hepatic congestion secondary to impaired venous return.

A 23-year-old male patient was admitted to our hospital on July 19, 2025, with the chief complaints of abdominal distension for 1 week and detection of a cardiac tumor 3 days prior to admission. The patient had a medical history of transcatheter atrial septal defect (ASD) occlusion 12 years earlier for congenital heart disease with ASD, with an uneventful postoperative recovery. One week before admission, he developed abdominal distension without obvious predisposing factors. A color Doppler ultrasound at a local hospital suggested right atrial space-occupying lesion (suspected myxoma) and tricuspid valve orifice obstruction, and he was admitted for further management under the tentative diagnosis of cardiac tumor. The patient denied a family history of hereditary diseases or tumors, and no similar cases were reported among his immediate relatives. On physical examination, vital signs were as follows: pulse rate 73 beats per minute, respiratory rate 17 breaths per minute, and blood pressure 107/71 mmHg. The cardiac rhythm was regular, and a tumor plop sound was auscultated at the cardiac apex. Post-admission auxiliary examinations yielded the following results:

Transthoracic echocardiography (Figure 1): An ASD occluder was visualized on the interatrial septum. A hyperechoic mass measuring approximately 63 × 43 mm was identified in the right atrioventricular cavity, with relatively clear borders, adherent to the right ventricular and atrial walls, low mobility, and causing obstruction of the right ventricular inflow tract; the tricuspid valve was poorly visualized. In the left atrium, a 38 × 28 mm hyperechoic mass with a 5-mm-wide pedicle attached to the root of the anterior mitral leaflet was observed, exhibiting high mobility and resulting in relative mitral stenosis. The echocardiographic diagnosis was: hyperechoic lesions in the right atrioventricular cavity and left atrium (space-occupying lesions obstructing the right and left ventricular inflow tracts, suspected myxoma); status post transcatheter ASD occlusion with resolution of interatrial shunt.
Electrocardiogram (ECG): Sinus rhythm, third-degree atrioventricular block, accelerated junctional rhythm, and mild T-wave changes.
PET-CT: Slightly hypodense space-occupying lesions in the heart with heterogeneous FDG uptake, suggestive of neoplasm (suspected myxoma); pericardial effusion; hepatomegaly with diffuse increased FDG metabolism; thickened and blurred mesentery.
Thoracoabdominal aortic CTA (Figure 2): Findings consistent with post-ASD occlusion status; pericardial effusion and right heart insufficiency; space-occupying lesions involving bilateral atria and ventricles (crossing the interatrial septum and occluder, suspected myxoma); bilateral pleural effusion with focal atelectasis of both lungs; heterogeneous hepatic enhancement (suspected hepatic congestion); hypoenhancing area in the uncinate process of the pancreatic head with blurred surrounding fat space (suspected pancreatitis); edematous and thickened gallbladder wall; abdominal and pelvic effusion.
Laboratory tests: Complete blood count: white blood cell count 13.45 × 10^9/L, hemoglobin 166 g/L, platelet count 107 × 10^9/L.
Blood biochemistry: alanine aminotransferase (ALT) 705.7 U/L, aspartate aminotransferase (AST) 521.9 U/L.
Tumor markers: carbohydrate antigen 125 (CA-125) 272.84 U/mL, neuron-specific enolase (NSE) 62.20 ng/mL.

After admission, the patient was administered pharmacological therapies including diuretics, hepatoprotective agents, and anti-infective medications. Routine laboratory tests and preoperative preparations were completed in anticipation of surgical intervention for the cardiac tumor. On the 5th day after admission, the patient experienced two episodes of transient amaurosis: The first episode occurred during coughing, lasting several seconds and resolving spontaneously without limb convulsions, post-episode discomfort, or incontinence of urine and feces. A second episode of amaurosis developed several hours later, with spontaneous resolution after approximately 1 min.

Emergency auxiliary examinations revealed the following abnormal results:

Arterial blood gas analysis: pH 7.436, partial pressure of carbon dioxide (PaCO₂) 24.9 mmHg, partial pressure of oxygen (PaO₂) 39.6 mmHg, actual bicarbonate (HCO₃^−^) 16.5 mmol/L, total carbon dioxide (TCO₂) 14.2 mmol/L, base excess (BE) -5.5 mmol/L, oxygen saturation (SaO₂) 67.4%, lactic acid 10.3 mmol/L.
Complete blood count: white blood cell count 18.96 × 10⁹/L, platelet count 16 × 10⁹/L, high-sensitivity C-reactive protein (hs-CRP) 45.77 mg/L.
Blood biochemistry: serum creatinine 151.5 μmol/L, blood urea nitrogen (BUN) 18.87 mmol/L, uric acid 978.0 μmol/L, total bilirubin 101.6 μmol/L, alanine aminotransferase (ALT) 2299.6 U/L, aspartate aminotransferase (AST) 1994.6 U/L, lactate dehydrogenase (LDH) 2736.0 U/L, creatine kinase (CK) 1349.0 U/L.
Brain natriuretic peptide (BNP): 1295 pg/mL.

Given the rapid disease progression and critical clinical status, the patient was transferred to the intensive care unit (ICU) for close monitoring and intensive supportive treatment. The cardiac surgery team conducted a discussion on this case and concluded that the patient's symptoms, signs, and laboratory abnormalities, including amaurosis fugax and acute liver failure, were caused by the rapid growth of the cardiac tumor obstructing the right ventricular inflow tract. The patient presented with acute hemodynamic compromise, with a strong indication for emergency surgery to relieve the obstruction, and no feasible alternative treatment was available. The patient's family was fully informed that the operation might only achieve palliative resection, with a high risk of tumor residual and postoperative recurrence. The family understood the situation and provided informed consent for the surgery.

**Figure 3:**
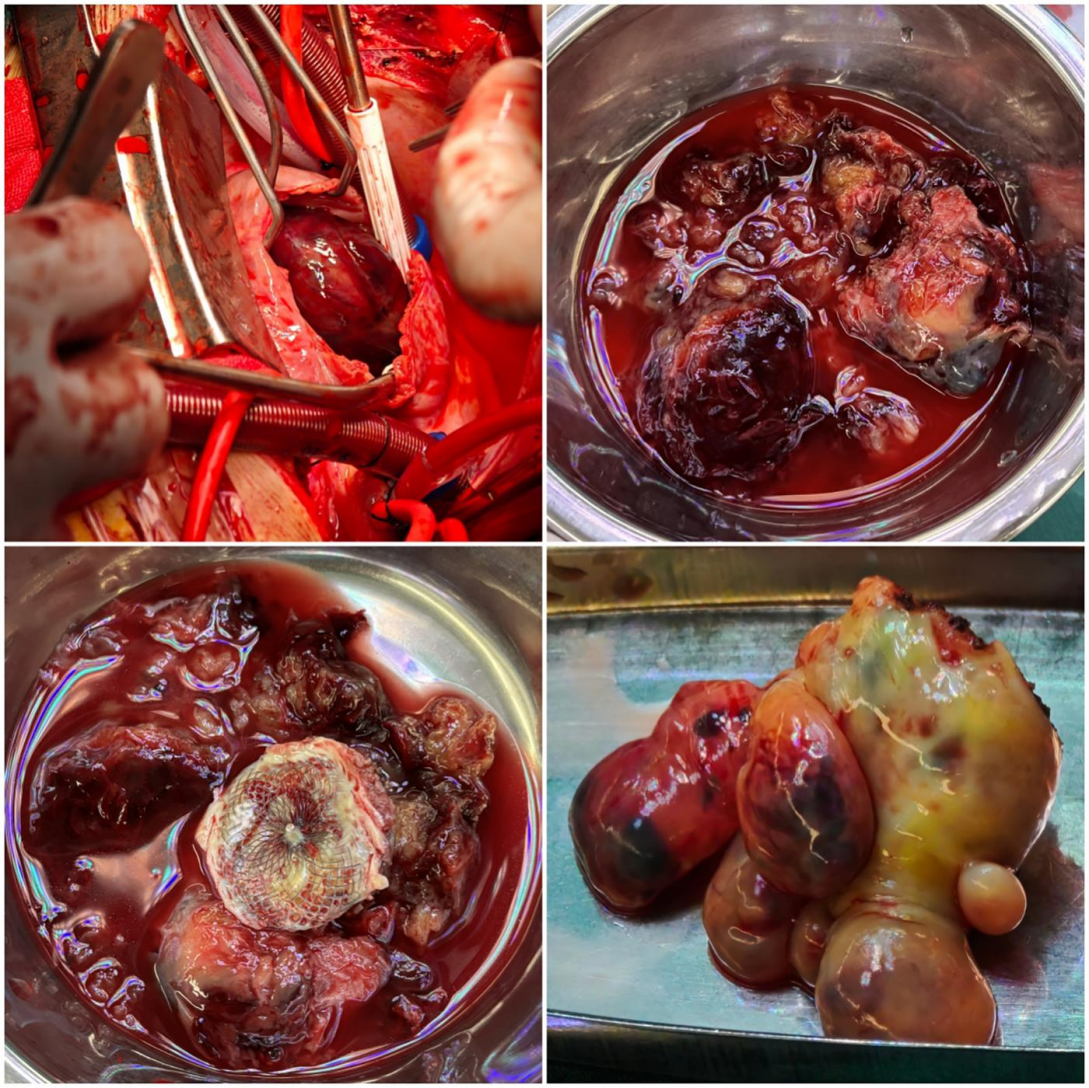
Pathological examination confirmed the diagnosis of cardiac myxosarcoma.

On the 6th day after admission, the patient underwent cardiac lesion resection. Intraoperative exploration via right atrial incision revealed that the tumor had a myxoid appearance, with its pedicle located at the junction of the interatrial septum and right atrioventricular groove and ill-defined borders. The tumor exhibited extensive involvement, protruding from the right atrium into the right ventricle with dimensions of approximately 5 cm × 6 cm × 6 cm; the tricuspid valve orifice was nearly completely obstructed by the tumor. A 3 cm × 4 cm ASD occluder was identified on the interatrial septum, with tumor tissue growing around its periphery and extending into the left atrium. The tumor had infiltrated the myocardium at the atrioventricular junction and the interatrial septum with a wide range of involvement, precluding complete resection. The majority of the tumor was resected (Figure 3) to relieve right cardiac circulatory obstruction, and the surgical wound was cauterized with an electrocautery device. Subsequently, the ASD occluder was removed (Figure 3), and the left atrium was accessed for exploration. A left atrial tumor measuring approximately 4 cm × 3.5 cm × 3 cm was found, with an ill-defined pedicle and invasive growth pattern. After tumor resection (Figure 3), the wound was cauterized with electrocautery, and the ASD was repaired using autologous pericardium. Following closure of the right atrial incision and cardiac reperfusion, bleeding was noted from the posterior wall of the left atrium. Despite repeated suturing for hemostasis, the bleeding persisted. Emergency re-establishment of cardiopulmonary bypass (CPB) was performed with systemic hypothermia, aortic cross-clamping, and cardioplegic infusion. Reopening of the left atrium revealed a 1 cm laceration on the posteroinferior wall, which was closed with continuous sutures. The heart was successfully resuscitated, and CPB was discontinued. Total operative duration was 345 min.

Morphological Features: Pathological examination of the left and right atrial masses revealed that the tumor cells were round to short spindle-shaped, arranged in solid sheets, fascicles, microcysts, and reticular patterns, with abundant myxoid stroma. The nuclei showed relatively uniform morphology and evenly distributed chromatin, with visible small nucleoli, and mitotic figures were relatively active. Pathological Diagnosis: Combined with the immunohistochemical results, the tumor was diagnosed as myxoid-rich mesenchymal neoplasm, with a primary consideration of soft tissue sarcoma (cardiac myxosarcoma). Pathological Note: The morphological features of this tumor are extremely rare, and the current immunophenotype failed to indicate its origin or differentiation. Given that the tumor cells exhibit a monotonous and uniform morphological pattern, it is suggested that there may be molecular abnormalities that can be used for diagnosis and classification. Targeted multi-gene RNA and DNA sequencing is recommended to assist in the definitive diagnosis. Considering financial constraints, the patient's family did not consent to further targeted multigene RNA and DNA sequencing.

The patient was admitted to the intensive care unit (ICU) for 6 days and to the general ward for 12 days after surgery. Symptoms such as abdominal distension were significantly improved compared with those before surgery, and the patient was discharged after a total hospitalization of 24 days. The last blood biochemistry test before discharge showed the following results: serum creatinine 67.5 μmol/L, blood urea nitrogen 5.67 mmol/L, alanine aminotransferase (ALT) 82.2 U/L, aspartate aminotransferase (AST) 34.7 U/L, and lactate dehydrogenase (LDH) 234.0 U/L, indicating that liver and kidney functions had basically returned to normal. After being fully informed of the clinical characteristics and prognosis of the disease, the patient's parents had been mentally prepared for the poor outcome. Furthermore, considering the treatment costs and other related issues, no anti-tumor pharmacotherapy was administered to the patient after surgery.

**Figure 4:**
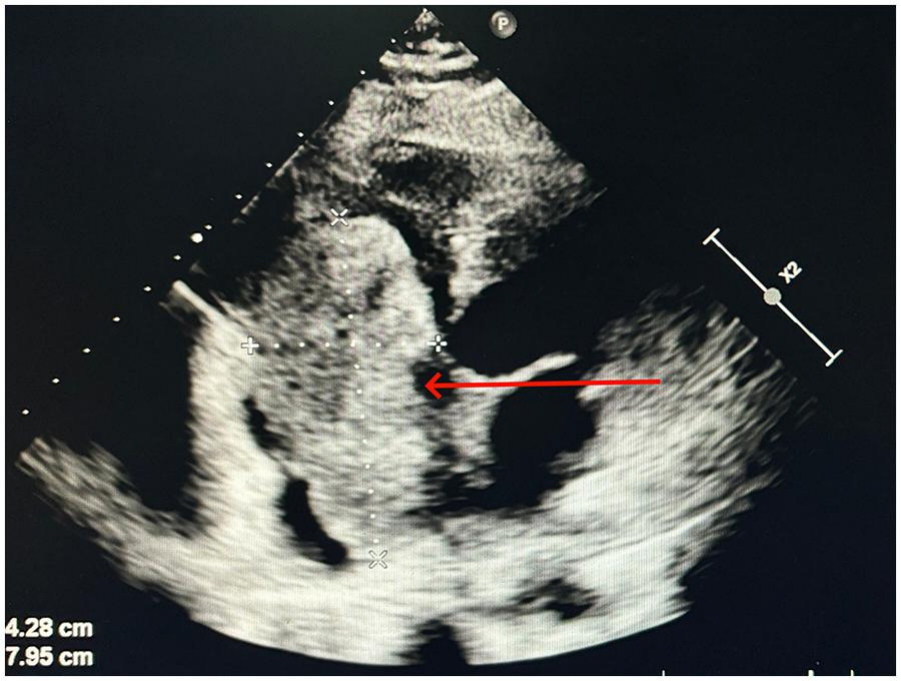
A giant mass was noted again in the right cardiac cavity, indicating tumor recurrence.

Approximately 50 days after surgery, the patient was readmitted to the hospital due to recurrent abdominal distension accompanied by nausea and vomiting. A follow-up transthoracic echocardiography revealed the following findings: a hyperechoic mass measuring approximately 74 × 42 mm was detected in the right atrioventricular cavity, which was adherent to the right ventricular wall and interatrial septum with low mobility, causing obstruction of the right ventricular inflow tract; the tricuspid valve was poorly visualized. In addition, a 13 × 11 mm hyperechoic mass was identified in the left atrium, attached to the inferior end of the interatrial septum (Figure 4). The echocardiographic diagnosis was hyperechoic lesions in the right atrioventricular cavity and left atrium (space-occupying lesions obstructing the right ventricular inflow tract), suggesting tumor recurrence. The patient died 2 months after surgery. The death diagnoses included cardiac myxosarcoma, cardiogenic shock, respiratory failure, liver failure, and renal failure.

## 3 Discussion

Primary malignant cardiac tumors are extremely rare and can occur at any age, with reported cases ranging from 3 months to 70 years old; approximately 75% of cases develop in patients under 49 years of age (2). The clinical manifestations of primary malignant cardiac tumors are heterogeneous, which depend on the tumor's location, distant metastasis, and embolic events, and can be summarized as follows: (1) Palpitations and dyspnea are the predominant symptoms, mainly resulting from valvular orifice obstruction, valvular damage, myocardial invasion by the tumor, and subsequent pericardial effusion or even cardiac tamponade. (2) Cardiac arrhythmias: Myocardial invasion by the tumor can induce atrial or ventricular arrhythmias, while conduction block caused by damage to the cardiac conduction system is relatively rare. (3) Vena cava obstruction. (4) Electrocardiographic manifestations mimicking myocardial infarction, which may be attributed to abnormal cardiac depolarization and repolarization secondary to myocardial infiltration by the tumor. (5) Syncope. (6) Other symptoms including chest pain, chest tightness, edema, irregular fever, anorexia, anemia, and weight loss.

Echocardiography is currently the most commonly used and crucial diagnostic tool for cardiac tumors, with high preoperative diagnostic accuracy. It can determine the tumor's location, size, shape, relationship with surrounding tissues, and the resulting hemodynamic changes, and also provides important reference value for distinguishing benign from malignant tumors. In the present case, the preoperative echocardiographic findings differed from those of typical benign myxomas: the space-occupying mass exhibited broad-based attachment to the atrial wall and interventricular septum (in contrast to the thin, short pedicle of benign myxomas) and showed relative stiffness, which was inconsistent with the soft texture of benign myxomas. As a non-invasive, convenient, and rapid examination, echocardiography has become a routine method for preoperative diagnosis and postoperative follow-up of cardiac tumors.

MRI is more suitable for comprehensively evaluating myocardial infiltration by tumors, intracavitary and extracavitary tumor extension, and differentiating cardiac tumors from paracardiac lesions. It can assist in distinguishing benign from malignant cardiac tumors; signs suggestive of malignancy include a large attachment area, involvement of multiple cardiac chambers, pericardium, or even extracardiac organs, and the presence of tumor necrosis (3). PET-CT can also be used to identify distant metastases and differentiate benign from malignant cardiac tumors. However, in the present case, the patient was unable to complete preoperative cardiac MRI due to intolerance to the examination.

Cardiac myxosarcoma most commonly occurs in the left atrium, with predominant infiltrative attachment to the left atrial wall (4, 5). Other sites of attachment include the mitral valve, interatrial septum, or a combination of these structures; in some cases involving the left atrium, extension to the pulmonary veins can be observed (4, 5). Reported cases have also documented involvement of both the left atrium and left ventricle (6), right atrium and right ventricle (7), and bilateral atria (8); pericardial involvement has also been described (5). In cases with bilateral atrial involvement, tumor extension to both ventricles and the main pulmonary artery may be present. Local recurrence can lead to infiltration of lung tissue and pericardial adipose tissue or other pericarial structures. Characteristically, the tumor is endocardially based, forming a homogeneous mass that protrudes into the atrial cavity (9).

Untreated cases of cardiac myxosarcoma typically result in short-term mortality. A variety of therapeutic modalities have been employed in the management of cardiac myxosarcoma, with the following approaches reported in the literature: (1) Surgical resection alone: A subset of cases is managed with isolated surgical tumor removal, which serves as a foundational intervention for localized lesions. (2) Autologous cardiac transplantation: Some patients have undergone autologous cardiac transplantation to achieve more extensive tumor resection in complex cases. (3) Combined modality therapy: A number of cases have received multimodal treatment integrating surgical resection with radiotherapy and/or chemotherapy to target residual tumor cells and reduce recurrence risk. (4) Adjuvant local ablation: For selected patients, cryoablation has been applied to the tumor's attachment site after resection as a prophylactic measure against local recurrence (10). (5) Orthotopic heart transplantation: Individual cases have undergone allogeneic heart transplantation for advanced or recurrent disease that cannot be adequately resected via conventional surgery (11). Among all therapeutic strategies for cardiac myxosarcoma, complete surgical resection is recognized as the most effective intervention, as it can significantly prolong patients' survival time (12). In particular, orthotopic heart transplantation, especially for left-sided cardiac tumors, increases the likelihood of achieving complete tumor extirpation and has been shown to extend patients' mean survival duration (13).

Transcatheter ASD occlusion is a well-established minimally invasive intervention for congenital ASD, yet it is associated with a spectrum of complications, which can be categorized as follows: (1) Vascular injury. (2) Embolism. (3) Residual shunt. (4) Arrhythmia. (5) Occluder displacement or detachment. (6) Aorto-left/right atrial fistula. (7) Pericardial effusion and cardiac tamponade. (8) Other rare complications: Rare adverse events include hemolysis, acute pulmonary edema, and infective endocarditis.

ASD occluders are primarily fabricated from materials such as medical nickel-titanium alloy and polytetrafluoroethylene, all of which have undergone rigorous biocompatibility verification. Post-implantation, these devices may only induce local inflammation and fibrosis (a type of foreign body reaction), and there is no evidence to indicate that they can trigger cellular carcinogenesis. The pathogenesis of cardiac myxosarcoma is mostly associated with genetic mutations and abnormal differentiation of mesenchymal tissue. To date, no definitive medical evidence has confirmed a direct causal association between transcatheter ASD occlusion and the occurrence of malignant cardiac tumors. Instead, the coexistence of these two conditions is more likely to be a pair of incidental independent events.

## 4 Conclusions

This article presents a rare case of a 23-year-old male patient with a history of transcatheter atrial septal defect (ASD) occlusion who was diagnosed with a giant bilateral atrial myxosarcoma. This case aims to provide broader clinical insights for readers regarding the decision-making and management strategies for aggressive and infiltrative malignant cardiac tumors. The primary goal of surgery is to relieve circulatory obstruction, rather than blindly pursuing complete resection. For tumors with infiltrative growth and non-curative potential, the benefits and risks of surgery should be carefully balanced, and the necessity of postoperative adjuvant therapy should be evaluated. Such tumors recur rapidly; close follow-up is required in the early postoperative period (1–3 months), with imaging studies used to detect signs of recurrence in a timely manner to avoid delays.

## Data Availability

The original contributions presented in the study are included in the article/Supplementary Material, further inquiries can be directed to the corresponding author.
