# Supplementary figures and images for: Case Report: Giant myxosarcoma involving both atria
Source: Front Surg. 2026 Apr 30;13:1765767. doi: 10.3389/fsurg.2026.1765767 (PMC13171733; doi:10.3389/fsurg.2026.1765767)

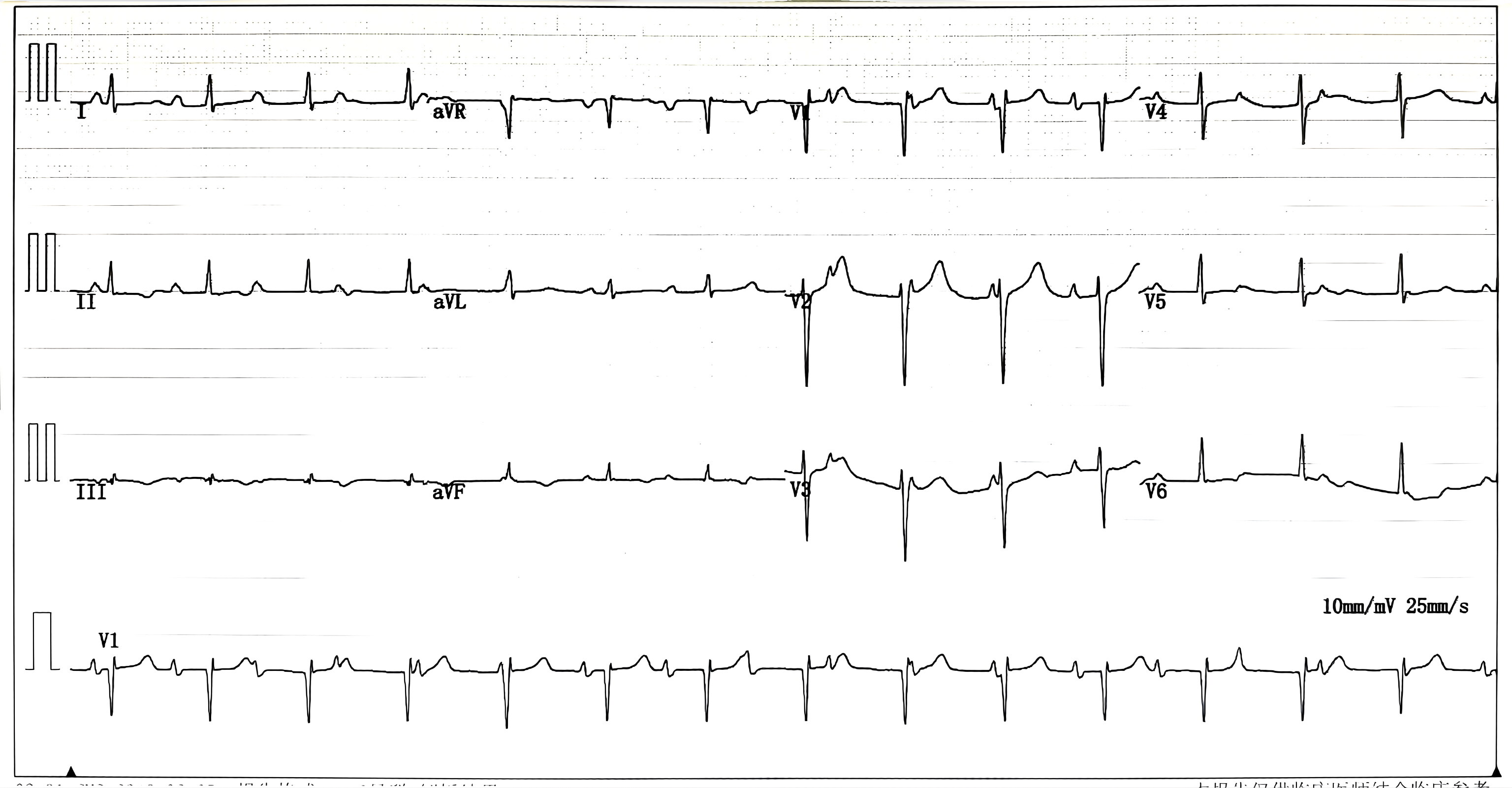

Supplement: Supplementary file 1 [file Image1.jpeg]

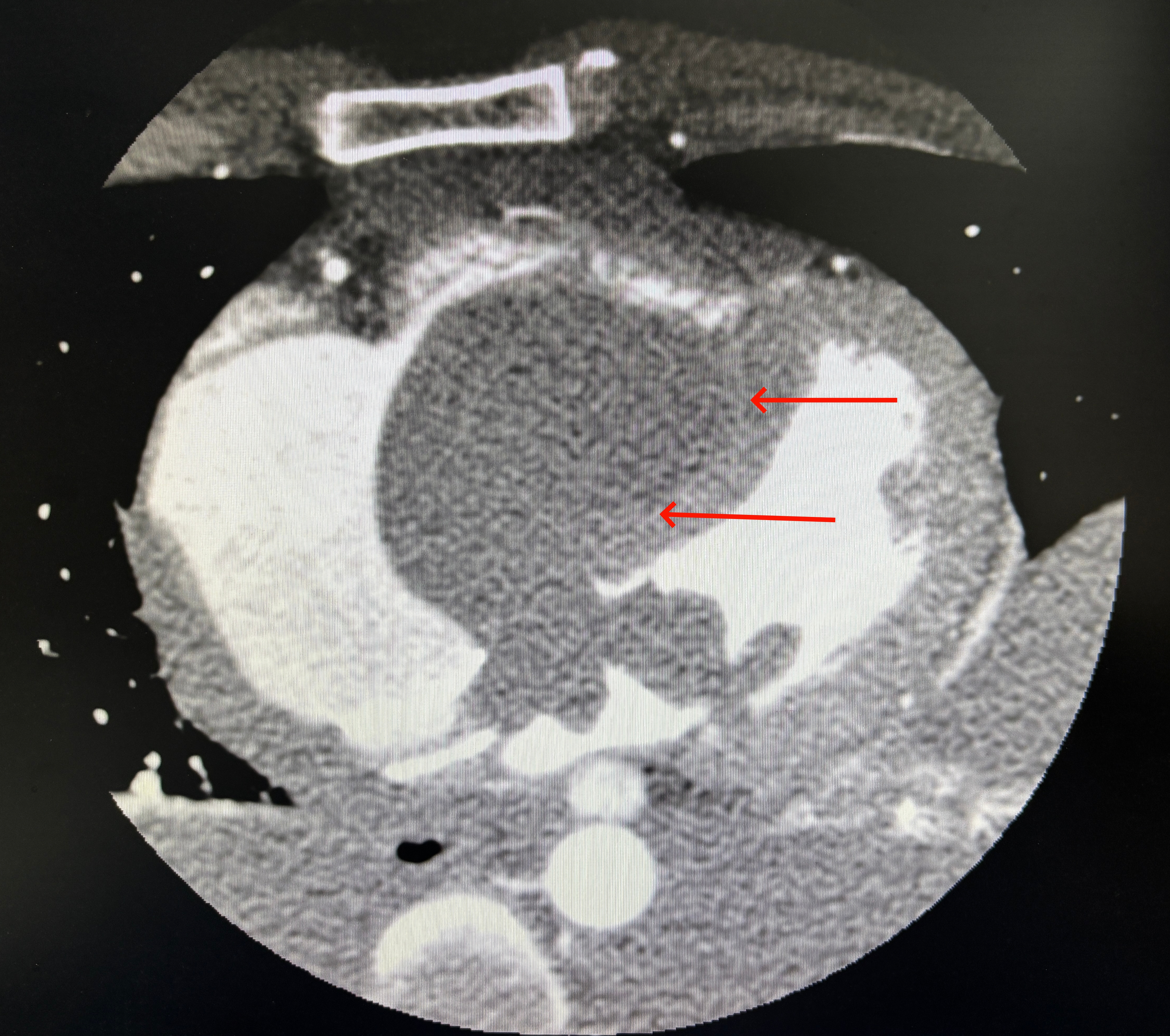

Supplement: Supplementary file 2 [file Image2.jpeg]

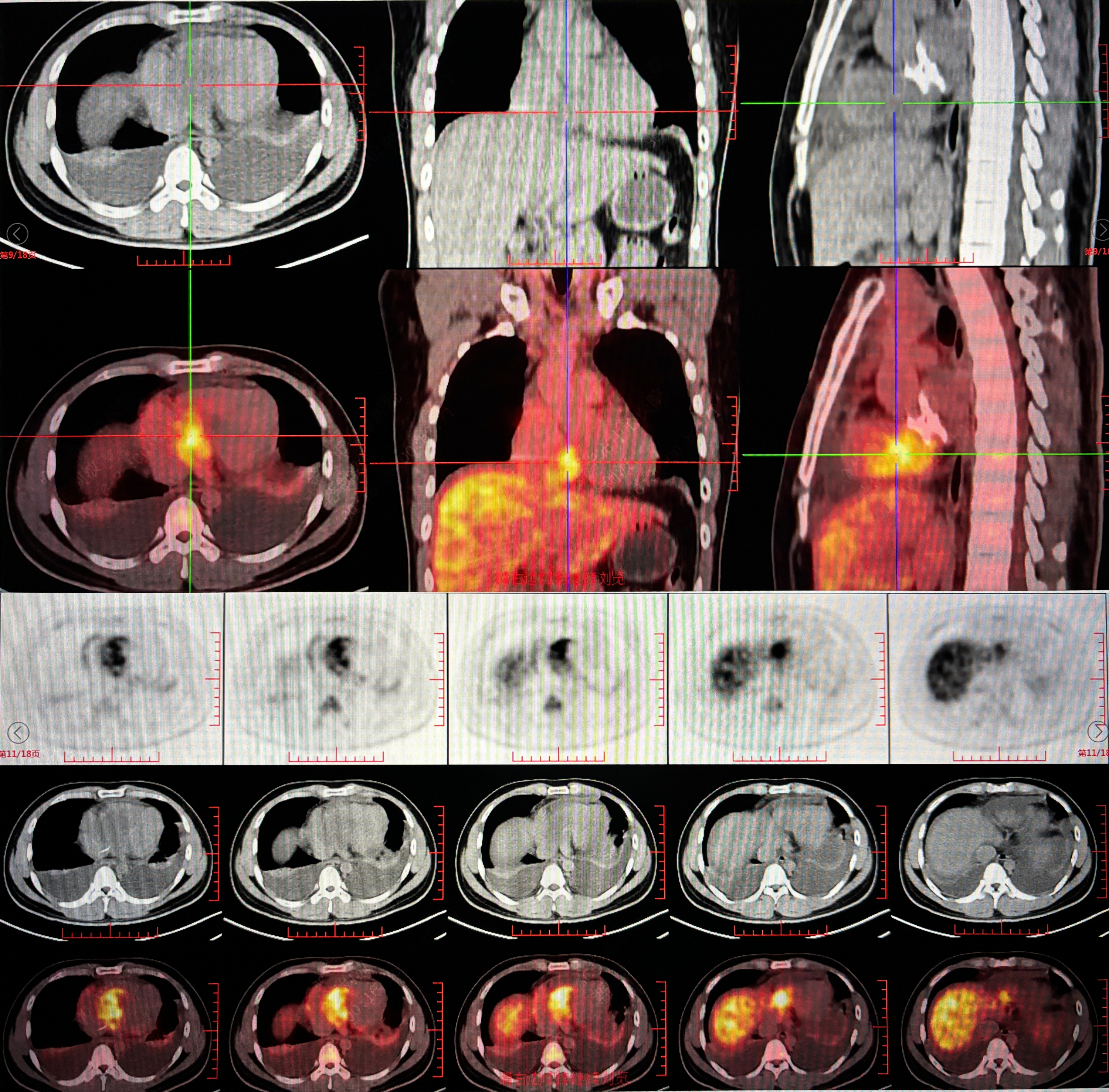

Supplement: Supplementary file 3 [file Image3.jpeg]
